# Ohio

**Published:** 1896-04

**Authors:** 


### OHIO.

The regular meetings of the State Board of Veterinary Examiners are held on the second Tuesday in April and the second Tuesday in July. The meetings this year fall on April 14th and July 14th. The meetings are held at the State Capitol at Columbus, in the Secretary of Agriculture's department, at 10 A.M. W. W. Miller succeeds Mr. Bonham in the State Board of Agriculture, and by virtue of his office is President of the Board of Examiners. Veterinarian N. B. Smith was appointed in the place of W. V. Lusk, resigned.

